# *Paragonimus kellicotti*: A Lung Infection in Our Own Backyard

**DOI:** 10.1155/2016/2107372

**Published:** 2016-04-24

**Authors:** Eric Johannesen, Van Nguyen

**Affiliations:** Department of Pathology and Anatomical Sciences, University of Missouri, Columbia, MO 65203, USA

## Abstract

Paragonimiasis is an infection caused by the lung fluke of the genus* Paragonimus*. Within the United States, paragonimiasis has been commonly diagnosed in Southeast Asian immigrants infected with the Asian lung fluke* Paragonimus westermani*. Infections from the North American lung fluke,* Paragonimus kellicotti*, have been rare, although more infections have been seen in people in the Midwestern United States. A 29-year-old male with a history of pleomorphic xanthoastrocytoma presented with hemoptysis. A CT scan showed a mass in the left upper lung lobe. A biopsy showed eosinophils and parasite eggs, some with a recognizable operculum. Further investigation revealed that he takes canoe trips on rivers within Missouri and would eat crayfish caught from these rivers. A blood sample was confirmed positive for Paragonimiasis serologically at the Center for Disease Control.* Paragonimus kellicotti* is found in rivers within the Mississippi basin. Infection occurs by consuming uncooked or undercooked crawfish. Microscopic identification of parasite eggs has been the gold standard. Serologic tests have been developed to aid in the diagnosis. Patients typically present with fever and hemoptysis. Common CT findings include pleural effusion, a mass, and lymphadenopathy. Awareness of* P. kellicotti* is important to guide appropriate diagnostic testing and ensuring proper treatment.

## 1. Introduction

Paragonimiasis is a disease caused by infection from a lung fluke of the genus* Paragonimus*.* Paragonimus* comprises 30 species with a global distribution. One species,* Paragonimus kellicotti*, is native to North America [1]. Cases of paragonimiasis within the United States have mostly been seen in immigrants or travelers to Southeast Asia due to infection by* Paragonimus westermani*, the Asian lung fluke. Although paragonimiases caused by* P. kellicotti* are rare, the numbers of people infected with this parasite have recently been increasing. To increase awareness that even native born Americans with no history of foreign travel can get this disease, we describe a case of a male infected by* P. kellicotti* from eating uncooked crayfish caught while vacationing on a river in Missouri.

## 2. Clinical Case

**Figure 1:**
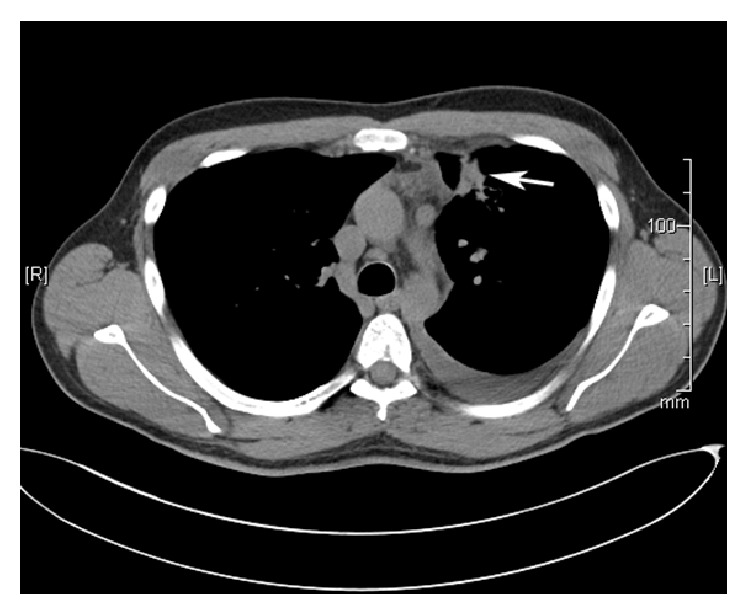
Chest CT showing the mass/area of consolidation in the left upper lobe (arrow).

**Figure 2:**
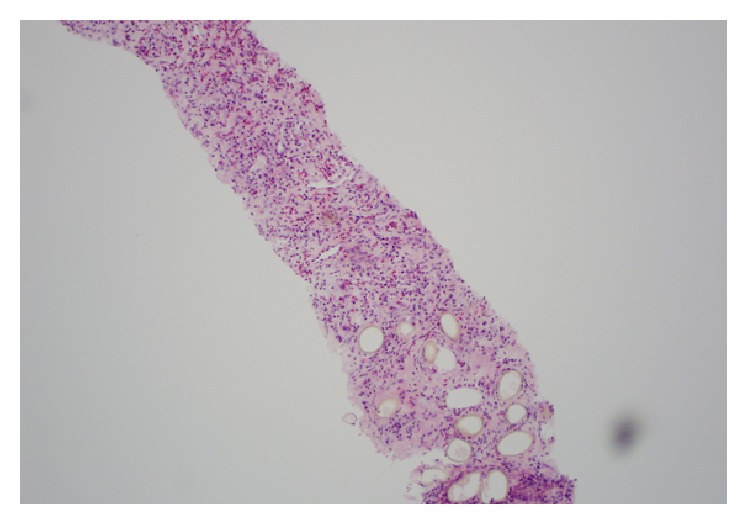
Low power view showing fibrosis with extensive eosinophils and parasitic eggs.

**Figure 3:**
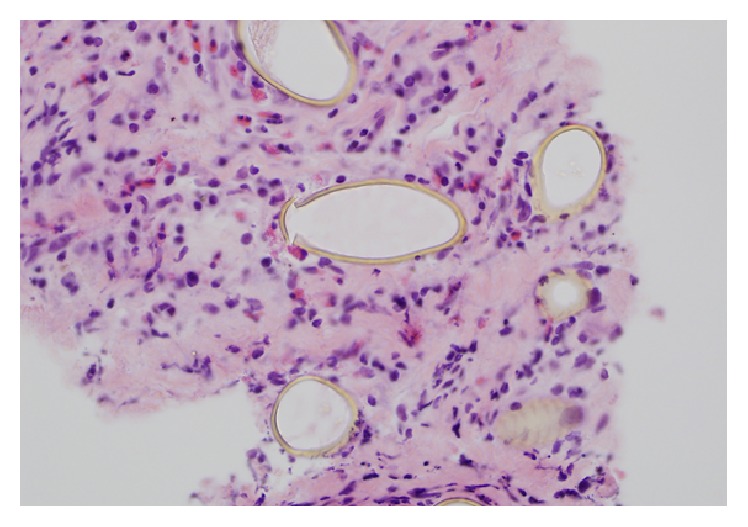
High power view showing an egg with an operculum (center).

**Figure 4:**
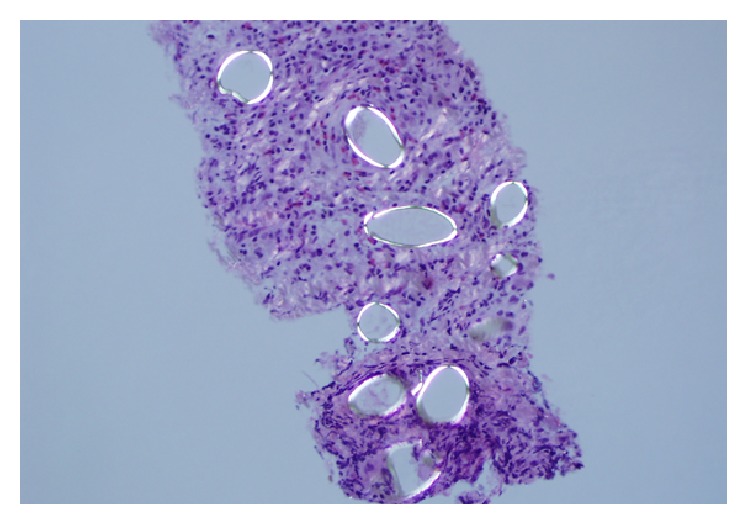
The eggs are birefringent on polarized light.

A 29-year-old male presented to his primary care physician with a complaint of hemoptysis that has been going on for a year. His past medical history was significant for pleomorphic xanthoastrocytoma that was treated with surgery in 1996 and 1999, radiation in 2001, and an additional excision in 2004. He works as a carpenter that renovates houses and, other than exposure to dust, denies any asbestos or chemical exposure. A complete blood count showed an elevated eosinophil count (0.9 × 10^−3^/mcl, normal range: 0.0–0.4 × 10^−3^/mcl). A CT scan showed a mass/area of consolidation in the left upper lobe of his lung along with mediastinal lymphadenopathy and a left pleural effusion (Figure 1). A bronchoscopy was nondiagnostic, so a CT guided biopsy was performed. The lung biopsy showed fibrosis with numerous eosinophils and several parasite eggs (Figure 2). Although most of the eggs were distorted, several had a recognizable operculum (Figure 3). The eggs were also birefringent on polarized light (Figure 4). These findings were consistent with a parasitic infection. Further investigation revealed that the patient takes yearly canoeing trips on the Current River in Missouri. During these trips, they frequently catch and eat boiled crayfish and drink alcohol. Although he stated that he never consumes raw crayfish, he did say that occasionally the canoe would tip over sending their cooked crayfish into the water, contaminating their food with uncooked crayfish. The morphologic features of the eggs in the biopsy coupled with a history of eating uncooked/undercooked crayfish were highly suggestive of paragonimiasis caused by* P. kellicotti*, the North American lung fluke. A blood sample was sent for* P. westermani* testing at the Center for Disease Control and the patient was treated with 25 mg/kg of Praziquantel three times per day for two days. The result of blood sample from the patient was subsequently confirmed positive for* P. westermani*. After one-month follow-up, a CT scan showed marked reduction in the size of the area of consolidation/mass in his left upper lung lobe. The patient also stated that he felt much better and had no new complaints.

## 3. Discussion

*Paragonimus kellicotti* is a species of lung fluke that is native to North America. It is found in streams and rivers within the Mississippi River basin, which includes the central United States between the Rocky and Appalachian mountains to the Gulf of Mexico. The* P. kellicotti* lifecycle starts with embryonated eggs that hatch into miracidia that invades snails (first intermediate host). They then emerge from snails as cercariae and invade crustaceans (second intermediate host) where they typically take up residence in the heart, becoming metacercariae. These infected crustaceans are eaten by mammals where the metacercariae penetrate the duodenum and traverse the diaphragm and into the parietal pleura and lung. The flukes then mature and lay unembryonated eggs within the bronchioles where they are carried to the upper airway where the eggs are either spit out or passed through the gastrointestinal system and deposited in the fecal matter [2, 3]. Direct visualization of the parasite eggs from sputum samples has been the traditional method of diagnosing paragonimiasis. However, serologic tests have been developed that detect antibodies produced by the host in response to the infection [1, 4].

Within the United States, paragonimiasis has most often been diagnosed in immigrants from Southeast Asia or travelers to the region where it is a common practice of consuming pickled freshwater crab, which may be infected with* P. westermani*, the lung fluke endemic to Southeast Asia. Recently, infections by the North American lung fluke,* P. kellicotti*, once considered rare, have been slowly increasing. Most of the infections have occurred in people who have caught and consumed crayfish in rivers within the state of Missouri, Oklahoma, and Ohio [4, 5]. One study examining the prevalence of* P. kellicotti* in crayfish found that out of 144 crayfish examined from three streams within Missouri 65% harbored metacercariae [3].

Paragonimiasis typically has a clinical presentation similar to an upper respiratory tract/lung infection. Symptoms typically include dyspnea, fever, cough, and hemoptysis. A complete blood count will frequently show an elevated eosinophil count. The CT finding most specific to paragonimiasis is a nodule with a line of opacification, most likely representing a worm tract, extending to the pleura. Other common imaging findings include pleural effusion, pleural thickening, a mass or consolidation, and mediastinal and/or hilar lymphadenopathy. On chest radiographs, the most common findings are pleural effusion, pulmonary infiltrates, and calcifications, but, in some cases (up to 40% in one study), no abnormality is seen [6, 7]. A very important clue is a history of consuming uncooked or undercooked crayfish caught in rivers within the Mississippi River basin where* P. kellicotti* is endemic [4]. Microscopic visualization of eggs in a sputum or stool sample is considered the gold standard for diagnosis, but they are not seen in all cases. Immunoassays that detect IgG antibodies against* P. westermani* and* P. kellicotti* antigens have been developed to confirm the diagnosis. The* P. westermani* assay, developed by the CDC, is 96% sensitive and 99% specific in diagnosing paragonimiasis [1]. A more recently developed assay utilizes antigens isolated from* P. kellicotti* and in limited studies has been more accurate in diagnosing North American paragonimiasis [8]. Newer techniques such as protein microarrays and DNA pyrosequencing are being developed to provide even more accurate and faster diagnoses [1].

## 4. Conclusion

*Paragonimus kellicotti* is the North American lung fluke that is endemic to rivers and streams within the Mississippi River basin. People who eat uncooked or undercooked crayfish infected with the parasite can develop paragonimiasis. The vast majority of* P. kellicotti* infections have occurred in people who have consumed crayfish caught in rivers in Missouri, Ohio, and Oklahoma. To ensure timely treatment, anyone who presents with hemoptysis, eosinophilia, fever, and suspicious infiltrates on imaging and has a history of consuming crayfish while taking camping or river trips in Midwestern rivers should be tested for paragonimiasis.
